# Integration of single-cell sequencing and machine learning identifies key macrophage-associated genetic signatures in lumbar disc degeneration

**DOI:** 10.3389/fimmu.2025.1671961

**Published:** 2025-12-02

**Authors:** Hongxing Zhang, Bin Dai, Jiafeng Peng, Minglei Gao, Danyang Li, Xiaowei Xiang, Junchen Zhu

**Affiliations:** 1 The Department of Orthopaedics, the Second Affiliated Hospital of Anhui University of Chinese Medicine, Hefei, Anhui, China; 2 The First Affiliated Hospital of University of Science and Technology of China, Hefei, Anhui, China; 3 Shenzhen Luohu District Hospital of Traditional Chinese Medicine, Shenzhen, Guangdong, China

**Keywords:** lumbar disc degeneration, single-cell RNA sequencing, machine learning, immune microenvironment, hdWGCNA

## Abstract

**Background:**

Lumbar disc degeneration, a primary cause of chronic low back pain, is closely linked to inflammatory responses and the immune microenvironment; however, its underlying mechanisms remain poorly understood.

**Methods:**

This study integrated scRNA-seq and bulk RNA-seq data to identify macrophage subpopulations in degenerative tissues and constructed co-expression modules using hdWGCNA. Functional enrichment was explored through GO, KEGG, and GSEA analyses. A panel of 101 machine learning algorithms was employed to screen diagnostic genes, with ROC curves used for validation. A combined diagnostic model for LDD risk was developed based on the expression profiles of the diagnostic genes. Additionally, immune infiltration was assessed via CIBERSORT, potential therapeutic compounds were identified and validated through molecular docking, and animal experiments were performed to verify the reliability of the results.

**Results:**

Single-cell analysis identified a pro-inflammatory macrophage subpopulation enriched in degenerative tissues. hdWGCNA revealed highly correlated black and blue modules, which were primarily associated with “immune signaling–matrix remodeling,” as indicated by enrichment analysis. Machine learning approaches screened key genes, including CDK1 and COL4A2, from these modules. ROC analysis confirmed the strong diagnostic performance of these genes, and the combined diagnostic model based on them demonstrated excellent predictive capability for LDD risk. Immune infiltration analysis highlighted a close association between the key genes and the γδT cell–neutrophil axis. Molecular docking suggested that RO 3306 and AR234960 may serve as potential therapeutic agents. qPCR and Western blot experiments validated the expression of the key genes and the possible effects of these compounds.

**Conclusion:**

This study elucidates the genetic signatures associated with macrophages and their immune regulatory mechanisms in LDD, identifies potential diagnostic biomarkers and therapeutic targets, and proposes new strategies for precision intervention.

## 1 Introduction

Lumbar disc degeneration (LDD) is one of the leading causes of chronic low back pain. Its prevalence rises substantially with age, severely compromising patients’ quality of life and representing a significant global public health concern. Epidemiological data indicate that approximately 80% of individuals experience some degree of low back pain during their lifetime, with LDD accounting for the majority of these cases (1).

Recent studies have increasingly highlighted the critical role of immune-inflammatory responses in the onset and progression of LDD, particularly the infiltration of immune cells—such as monocytes, macrophages, and T cells—into the nucleus pulposus region (2). Macrophages contribute to extracellular matrix (ECM) degradation and sensitize pain pathways through the secretion of pro-inflammatory cytokines (e.g., IL-1β, TNF-α) and matrix metalloproteinases (e.g., MMP-9, MMP-13). Consequently, they are considered key regulatory factors in the early stages of LDD (3). However, immune cells within LDD tissues exhibit substantial heterogeneity, and the functions and regulatory mechanisms of various subpopulations at different stages remain incompletely understood. This knowledge gap has hindered the advancement of immune-targeted therapies for LDD.

Single-cell RNA sequencing (scRNA-seq) has recently been applied extensively to the study of various tissues and diseases, providing single-cell resolution for dissecting the cellular composition, states, and trajectories within tissues. This technology offers new opportunities to investigate immune heterogeneity in LDD (4). At the same time, bulk RNA sequencing remains a pivotal tool in clinical subgroup analyses, differential gene expression studies, and the estimation of immune infiltration. Integrating scRNA-seq with bulk RNA-seq data not only allows precise identification of critical cellular subpopulations but also validates candidate genes at the tissue level, representing a key direction in multi-omics research (5). Moreover, machine learning has emerged as an effective tool for identifying diagnostic biomarkers and developing predictive models from high-dimensional transcriptomic data. Algorithms such as LASSO, SVM, and random forests enhance the accuracy and generalizability of feature selection for gene prediction (6). By integrating public databases with molecular docking approaches, potential therapeutic targets among existing compounds can be screened, thereby accelerating the development of targeted interventions (7).

Therefore, this study aims to systematically analyze the immune microenvironment of degenerative nucleus pulposus tissues by integrating scRNA-seq and bulk RNA-seq data. We construct co-expression modules based on a weighted co-expression network and explore their biological significance through pathway enrichment analyses. Machine learning algorithms are employed to identify diagnostic genes and establish diagnostic models. Additionally, immune infiltration analysis and drug screening are combined to uncover novel immune regulatory mechanisms in LDD and to identify molecular targets with diagnostic and therapeutic potential, thereby providing a theoretical foundation for precision treatment of LDD.

## 2 Materials and methods

### 2.1 Data acquisition

Data were retrieved from the GEO database (https://www.ncbi.nlm.nih.gov/geo/), including scRNA-seq data from GSE244889 and bulk RNA-seq data from GSE124272 and GSE23130 for downstream analyses (8, 9). In total, 17 LDD samples and 18 control samples were included: GSE244889 comprised 3 LDD and 4 control samples, GSE124272 included 8 LDD and 8 controls, and GSE34095 contained 6 LDD and 6 controls. Additionally, GSE124272 was designated as the training set, and GSE34095 as the validation set. Clinical phenotype information was also retrieved.

### 2.2 Single-cell RNA sequencing data processing and cell annotation

The raw scRNA-seq data were processed using the standard workflow of the Seurat package (v4.0) in R. Quality control criteria were set as follows: each cell was required to express at least 200 genes, and the proportion of mitochondrial gene expression could not exceed 20%. Batch effects were corrected using the Harmony algorithm (v0.1.0) following normalization and identification of highly variable genes. Subsequently, principal component analysis (PCA) and UMAP were performed for dimensionality reduction and clustering to obtain robust cell subpopulation classifications. Clusters were annotated based on canonical marker genes reported in the literature to identify major cell types, including nucleus pulposus and immune cells. Based on the initial clustering results, immune cells were further re-clustered for detailed subpopulation analysis. The polarization states of macrophage subtypes were identified according to the expression of canonical marker genes: M1-type macrophages were characterized by high expression of *CD86*, *NOS2*, *IL1B*, *TNF*, and *CXCL10*; M2-type macrophages by high expression of *CD163*, *CD206 (MRC1)*, *ARG1*, *IL10*, and *TGFβ1*; and unpolarized (M0) macrophages by low expression of both M1 and M2 signature markers. These classifications served as the basis for downstream functional analyses of macrophage subpopulations (10, 11).

### 2.3 High-dimensional weighted gene co-expression network analysis

Based on the previously identified macrophage subpopulations (M1, M2, and unpolarized types), this study applied high-dimensional weighted gene co-expression network analysis (hdWGCNA, v0.2.0) to identify key gene modules associated with lumbar disc degeneration. The expression matrix of macrophage subsets was extracted from the Seurat object and normalized using variance-stabilizing transformation (VST). A hierarchical clustering tree of genes was constructed using a dynamic hybrid cutting algorithm, with the minimum module size set to 30 genes. Module eigengenes (MEs) were then calculated to represent the overall expression profile of each module, and module detection was optimized by setting the dynamic cut parameter to deepSplit = 2. The soft-thresholding power was determined according to the scale-free topology criterion (R² > 0.8) to ensure conformity to scale-free network properties. Subsequently, correlations between module eigengenes and the degeneration phenotype (degenerative vs. normal groups) were computed to identify significantly associated modules (|R| > 0.5, p < 0.05, FDR-adjusted). The spatial expression patterns of these modules were further validated by UMAP mapping in Seurat v4.0. Hub genes within each module were defined as those with module membership (kME) > 0.8, indicating a Pearson correlation coefficient greater than 0.8 with the module eigengene and implying a core regulatory role within the module. Finally, hub genes from the key modules were subjected to Gene Ontology (GO) and Kyoto Encyclopedia of Genes and Genomes (KEGG) enrichment analyses to elucidate the critical regulatory networks underlying macrophage polarization during LDD (12).

### 2.4 Functional enrichment analysis and GSEA

The clusterProfiler package (version 4.0) was used to perform GO and KEGG pathway enrichment analyses on the screened hub genes, revealing their biological functions (13). Gene set enrichment analysis (GSEA) was conducted to determine whether predefined gene sets were enriched at the top or bottom of the ranked gene list, indicating upregulation or downregulation of the corresponding genes. The GSEA R package was applied to cluster the hub gene expression data and to assess enrichment relationships with key pathways (e.g., HALLMARK, Reactome), thereby evaluating potential mechanisms.

### 2.5 Machine learning feature selection and diagnostic model construction

In this study, a total of 101 machine learning models were assessed for diagnostic performance on the gene expression datasets. Five-fold cross-validation (5-fold CV) was employed to select the top five models with the highest area under the receiver operating characteristic curve (AUC). Subsequently, feature importance analysis based on the Random Forest (RF) algorithm was performed to identify a set of key biomarker genes (importance score > 20). For the XGBoost model, hyperparameter optimization was conducted with a focus on tuning core parameters, including the learning rate, maximum tree depth, and number of boosting rounds. In an independent validation dataset, the robustness of the top-performing models (rf, xgbTree, and ranger) was further confirmed through ROC curve analysis and DeLong’s test. Additionally, a two-layer Stacking ensemble model was constructed, integrating the rf and xgbTree algorithms, with logistic regression employed as the meta-classifier. All models were trained using an identical feature space, and performance was evaluated using AUC, F1-score, and accuracy as key metrics. To prevent overfitting, early stopping and nested cross-validation (nested CV) strategies were implemented. Finally, prediction probability distribution analysis and SHAP value interpretation were applied to elucidate the contribution patterns of key genes to model predictions (14, 15).

### 2.6 Evaluation of diagnostic performance of hub genes

To assess the diagnostic potential of the identified hub genes in distinguishing LDD samples from controls, we first extracted the expression matrices of these target hub genes in the training set and visualized differential expression using box plots. Wilcoxon rank-sum tests were conducted to determine significance levels, which were indicated with asterisks. Subsequently, ROC curves were generated using the pROC package, with gene expression values as predictors and group labels as response variables. AUC values were calculated for each gene, and ROC curves were plotted to evaluate diagnostic accuracy (16). The same procedure was applied to the validation set for confirmation.

### 2.7 Nomogram construction, model calibration, and decision curve analysis

A combined diagnostic model was constructed by integrating the expression profiles of CDK1 and COL4A2. A logistic regression model was constructed using the rms package, and a nomogram was plotted to visualize the relative contributions of each gene to disease risk. Model performance was evaluated with the Hosmer-Lemeshow test (g = 10) and bootstrap-based calibration (1,000 resamplings) to assess fit and predictive accuracy. To further evaluate clinical applicability, decision curve analysis (DCA) was performed. The Firth correction was applied to mitigate small-sample bias, and 100 bootstrap replicates were used to calculate confidence intervals of the net benefit, allowing for a comparison of the clinical utility between the joint model and single-gene models across various decision thresholds (17).

### 2.8 Immune cell infiltration analysis

CIBERSORT, a widely used method based on linear support vector regression for deconvolving immune cell expression matrices, was applied to quantify infiltrating immune cell subsets using bulk RNA-seq data. Proportions of immune cell subsets, including macrophages, T cells, and NK cells, were compared between LDD and control groups. Additionally, the correlation between key gene expression and specific immune cell subpopulations was analyzed to explore potential immunoregulatory roles. The reliability of the CIBERSORT results was further validated using the xCell algorithm (18).

### 2.9 Drug target screening and molecular docking

The PubMed database was searched using the keywords “CDK1” and “COL4A2” to identify potential therapeutic agents for these conditions. Molecular docking analyses were performed using AutoDock Vina to model the binding interactions between key proteins (CDK1, COL4A2) and candidate drugs (AR234960, RO 3306), and binding energies and molecular compatibility were evaluated (19).

### 2.10 Reagents

Cell culture media and related reagents: α-MEM medium – Gibco (Thermo Fisher Scientific, Waltham, MA, USA); DMEM medium – Gibco (Thermo Fisher Scientific, Waltham, MA, USA); DMEM/F12 medium – Gibco (Thermo Fisher Scientific, USA); fetal bovine serum (FBS) – Gibco (Thermo Fisher Scientific, USA); penicillin–streptomycin solution – Gibco (Thermo Fisher Scientific, USA); CCK-8 assay kit – Dojindo Laboratories (Kumamoto, Japan); collagenase type II – Sigma-Aldrich (Merck KGaA, Germany).

Reagents for molecular biology and protein experiments: EZ-press RNA Purification Kit – EZBioscience (B0004D, USA); PrimeScript RT Reagent Kit – TaKaRa (RR037A, Japan); SYBR Green qPCR Master Mix – TaKaRa (RR420A, Japan); RIPA lysis buffer containing protease and phosphatase inhibitors – Thermo Fisher Scientific (USA); 10% SDS-PAGE gel – Cwbiotech (Beijing, China); PVDF membrane – Thermo Fisher Scientific (USA); blocking buffer – Epizyme (Shanghai, China); ECL detection reagent (KF8003) – Affinity (Jiangsu, China).

Antibodies: COL4A2 (Collagen Type IV Alpha 2 Chain, 1:1000) – Proteintech (#14695-1-AP, RRID: AB_10699877); CDK1 (Cyclin-Dependent Kinase 1, 1:1000) – Proteintech (#19532-1-AP, RRID: AB_2881388); MMP3 (Matrix Metalloproteinase 3, 1:1000) – Proteintech (#17873-1-AP, RRID: AB_2138307); SRGN (Serglycin, 1:1000) – Thermo Fisher Scientific (#PA5-113692, RRID: AB_2884207); HRP-conjugated anti-mouse IgG (H+L, 1:5000) – Proteintech (#SA00001-1, RRID: AB_2722565); HRP-conjugated anti-rabbit IgG (H+L, 1:5000) – Proteintech (#SA00001-2, RRID: AB_2722564).

### 2.11 Isolation and culture of primary nucleus pulposus cells

Male C57BL/6J mice aged 4–6 weeks were obtained from SPF (Beijing) Biotechnology Co., Ltd. (license number: SCXK [Beijing] 2021-0011). All experimental protocols were approved by the Ethics Committee of the Laboratory Animal Center, The First Affiliated Hospital of the University of Science and Technology of China (approval No. 2023-N(A)-183, May 2023). After euthanasia, the mouse caudal vertebrae were immediately placed in pre-cooled sterile PBS. Under a dissecting microscope, intervertebral disc tissues were isolated, and nucleus pulposus tissues were carefully dissected and collected. The tissues were digested in 0.2% collagenase type II at 37 °C with gentle agitation for 4–6 hours. Following digestion, cells were collected by centrifugation (1000 rpm, 5 min), and the supernatant was discarded. The cell pellet was resuspended in DMEM/F12 complete medium supplemented with 10% FBS and 1% penicillin–streptomycin, and cultured at 37 °C in a humidified atmosphere containing 5% CO_2_. After cell attachment, non-adherent cells were removed by replacing with fresh medium. When cells displayed stable morphology and healthy growth, they were passaged, and second to third passages (P2–P3) were used for all subsequent experiments to ensure phenotypic and functional consistency.

### 2.12 Determination of optimal working concentrations of drugs

To determine the optimal effective concentrations of the CDK1 inhibitor RO 3306 and the candidate compound AR234960, primary nucleus pulposus cells were subjected to a cell viability assay (CCK-8 method). Cells were seeded in 96-well plates at a density of 5 × 10³ cells/well. After attachment, the medium was replaced with fresh medium containing different drug concentrations: RO 3306 group: 0, 1, 2.5, 5, 7.5, and 10 µM; AR234960 group: 0, 10, 25, 50, 75, and 100 µM. Each concentration was tested in six replicate wells, with blank controls included. After 24 hours of drug treatment, 10 µL of CCK-8 reagent was added to each well and incubated for an additional 2 hours. Absorbance was measured at 450 nm (OD_450_). Cell viability and inhibition rates were calculated, and dose–response curves were plotted. The concentration maintaining >70% cell viability (corresponding to 20%–30% inhibition) was defined as the optimal working concentration for subsequent pharmacodynamic studies.

### 2.13 IL-1β–induced degenerative model of nucleus pulposus cells and drug intervention

To mimic the inflammatory microenvironment of intervertebral disc degeneration and to evaluate drug efficacy, an IL-1β–induced degeneration model of nucleus pulposus cells was established. The experiment included four groups (n = 3 per group):

1. Control group: complete culture medium;
2. Model group (IL-1β): complete medium + 10 ng/mL IL-1β;
3. RO 3306 treatment group: complete medium + 10 ng/mL IL-1β + RO 3306 (optimal concentration);
4. AR234960 treatment group: complete medium + 10 ng/mL IL-1β + AR234960 (optimal concentration).

Cells were seeded in 6-well plates or culture dishes, and once cell confluence reached 70%–80%, the medium was replaced according to the experimental grouping. All treatments were maintained for 24 hours. After treatment, both cells and supernatants were collected for subsequent molecular assays (Western blot analysis).

### 2.14 Annulus fibrosus puncture model

Thirty adult male Sprague-Dawley (SD) rats (≥12 weeks old, 230–300 g) were obtained from Hangzhou Ziyuan Laboratory Animal Technology Co., Ltd. (Hangzhou, China; License No.: SCXK 2019-0004). The sample size was calculated using G*Power software (version 3.1), with the significance level (α) set at 0.05, the effect size (d) at 0.8, and the statistical power at 0.82, meeting the statistical requirements for animal experiments. Animals were housed under controlled temperature and humidity with free access to food and water. Under sevoflurane anesthesia, rats were fixed, and the Co5/Co6 (caudal vertebrae) or L5/L6 (lumbar vertebrae) segments were selected. Under X-ray guidance, a 21G puncture needle was inserted percutaneously, penetrating the full thickness of the annulus fibrosus into the nucleus pulposus (approximately 2–3 mm depth). The needle was rotated 180° and left in place for 30 seconds to induce controlled injury. Penicillin was administered intramuscularly postoperatively to prevent infection. Two weeks after surgery, intervertebral disc height was assessed via X-ray imaging to evaluate the success of the model induction (20). Western blotting (WB) and quantitative PCR (qPCR) were performed on sham, 1-month, 2-month, and 3-month postoperative groups to measure the expression levels of CDK1 and COL4A2. All experimental protocols were approved by the Animal Ethics Committee of the First Affiliated Hospital of the University of Science and Technology of China (Approval No.: 2023-N(A)-183, May 2023).

### 2.15 Evaluation of potential drug efficacy

To evaluate the therapeutic efficacy of potential drugs for lumbar disc degeneration, an SD rat model of LDD was established. Twelve rats were randomly assigned to four groups (n = 3 per group): control (saline), RO 3306 monotherapy (5 mg/kg, once weekly), AR234960 monotherapy (500 mg/kg, twice daily), and combination therapy. The drug administration protocol was as follows: the AR234960 group received intraperitoneal injections twice daily (with a 12-hour interval), the RO 3306 group received intraperitoneal injections once weekly, and the combination group received both agents at the same doses but with staggered administration. All treatments were continued for 4 weeks. After treatment, spinal cord tissues were collected from each group, and the expression levels of LDD-associated markers, MMP3 and SRGN, were examined by Western blot and qPCR (21, 22).

### 2.16 Quantitative real-time PCR

Total RNA was extracted using the EZ-press RNA Purification Kit, and 1 μg of RNA was reverse-transcribed into cDNA with the PrimeScript RT Reagent Kit. Quantitative PCR was performed using SYBR Green Master Mix, with three technical replicates for each sample. Amplification was carried out on an ABI 7500 Real-Time PCR System (Applied Biosystems, USA). Relative gene expression was calculated using the 2^−ΔΔCt method, with GAPDH as the internal reference. The sequences of the primers used are listed in **Supplementary Table 1**.

### 2.17 Western blotting

Samples were lysed in RIPA buffer containing protease and phosphatase inhibitors and incubated on ice for 15 minutes. Lysates were centrifuged at 14,000 × g for 5 minutes at 4°C, and the supernatants were collected. Protein concentrations were determined using a BCA Protein Assay Kit. Equal amounts of protein were mixed with 5× SDS-PAGE loading buffer, boiled for 5 minutes, and separated by 10% SDS-PAGE, followed by transfer onto PVDF membranes. Membranes were blocked with 5% non-fat milk for 1 hour and then incubated overnight at 4°C with primary antibodies. The next day, membranes were incubated with HRP-conjugated secondary antibodies for 2 hours at room temperature. Protein bands were visualized using an ECL detection reagent and imaged with a chemiluminescence imaging system (Proteinsimple FluorChem R, USA). Band intensities were quantified using ImageJ software (NIH, USA).

### 2.18 Statistical analysis

Data differences were analyzed using unpaired t-tests or the Wilcoxon rank-sum test, as appropriate. Pearson correlation analysis was performed to assess correlations in tissue expression levels. All statistical tests were two-tailed, and the Benjamini–Hochberg method was applied to control the false discovery rate (FDR) for multiple comparisons. A p-value < 0.05 was considered statistically significant. All analyses were conducted using R software (version 4.1.3).

## 3 Results

### 3.1 Identification of LDD cell clusters

**Figure 1:**
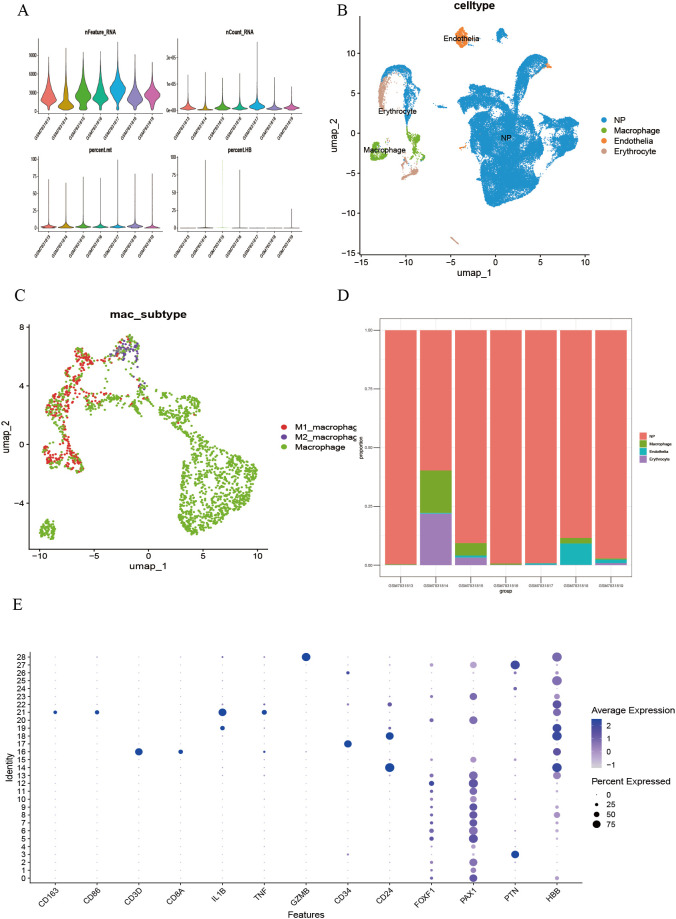
Single-cell transcriptomic analysis identifies cell clusters in LDD. **(A)** Quality control and normalization results of multiple single-cell batches; **(B)** Identification and UMAP spatial distribution of significant cell populations; **(C)** Fine classification of macrophage functional subsets; **(D)** Quantitative analysis of cellular composition heterogeneity across samples; **(E)** Validation of cell type-specific marker expression.

Single-cell transcriptome analysis was performed on the GSE244889 dataset. After excluding low-quality cells based on quality control (QC) standards, the overall QC metrics indicated good data quality (nFeature_RNA approximately 500 ± 50, nCount_RNA distributed within 400–700), with no significant batch effects observed (**Figure 1A**). Dimensionality reduction and clustering of the processed cells using the UMAP algorithm identified 28 cell clusters (**Supplementary Figure 1**). By annotating the clustering results with classical marker genes retrieved from the literature (**Figure 1E**), four significant core cell populations were identified: endothelial cells, macrophages, erythrocytes, and neural progenitor cells, each showing distinct boundaries in the UMAP distribution (**Figure 1B**). Macrophages were further subdivided into pro-inflammatory M1, anti-inflammatory M2, and unpolarized (M0) subgroups. The observed continuum from M1 to M2 indicates a dynamic polarization state within the microenvironment (**Figure 1C**). Sample composition analysis showed that macrophages were significantly enriched in degenerative samples such as GSM5703913, GSM5703915, and GSM5703918 (**Figure 1D**).

### 3.2 Identification of immune-cell-associated modules by hdWGCNA

**Figure 2:**
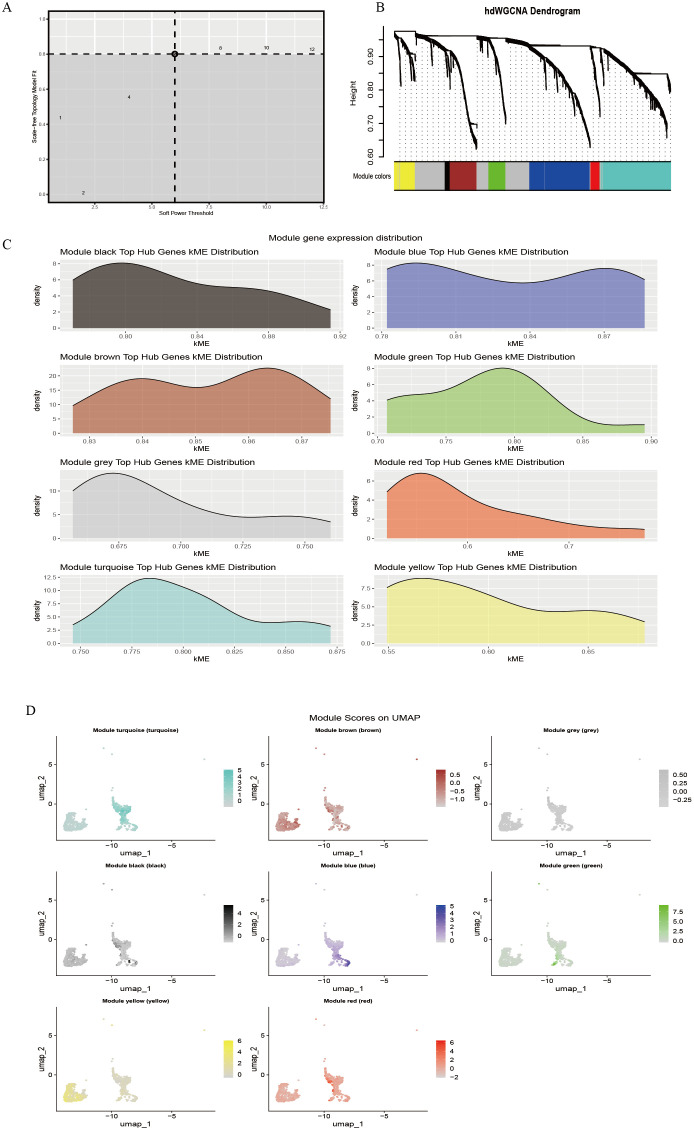
hdWGCNA identifies immune cell-related gene modules. **(A)** Soft-threshold power selection analysis; **(B)** Gene co-expression dendrogram; **(C)** Distribution of module hub genes; **(D)** Spatial localization map of modules.

The hdWGCNA method was applied to construct a disease-associated regulatory network. First, the soft threshold (β) was determined based on the scale-free topology criterion, and at β = 5, the scale-free topology fit exceeded 0.8 (dashed threshold), meeting the assumptions of a scale-free network (**Figure 2A**). Under this parameter, an adjacency matrix was constructed and transformed into a topological overlap matrix (TOM). Hierarchical clustering identified eight co-expression modules (**Figure 2B**), represented by turquoise, blue, brown, green, yellow, red, black, and gray. The module clustering height ranged from 0.60 to 0.90, with the black module (cluster height > 0.85) exhibiting the strongest cohesiveness. Module membership (kME) analysis indicated variable co-regulation strength among modules (**Figure 2C**). The black module (kME = 0.80–0.92) and blue module (kME = 0.78–0.87) exhibited a concentrated distribution, suggesting strong gene co-regulation within each module; the yellow (kME = 0.55–0.65) and red modules (kME = 0.60–0.70) displayed relatively dispersed distributions, indicating potential functional heterogeneity or participation in multiple pathways. Further spatial expression mapping (**Figure 2D**) showed that the turquoise module was widely distributed across the UMAP space (approximately −2 to 2), the brown module was enriched in specific cell clusters, and the gray module (expression range −0.25 to 0.5) showed low-level diffuse expression, consistent with background gene characteristics.

### 3.3 Functional enrichment of genes in the blue and black modules

**Figure 3:**
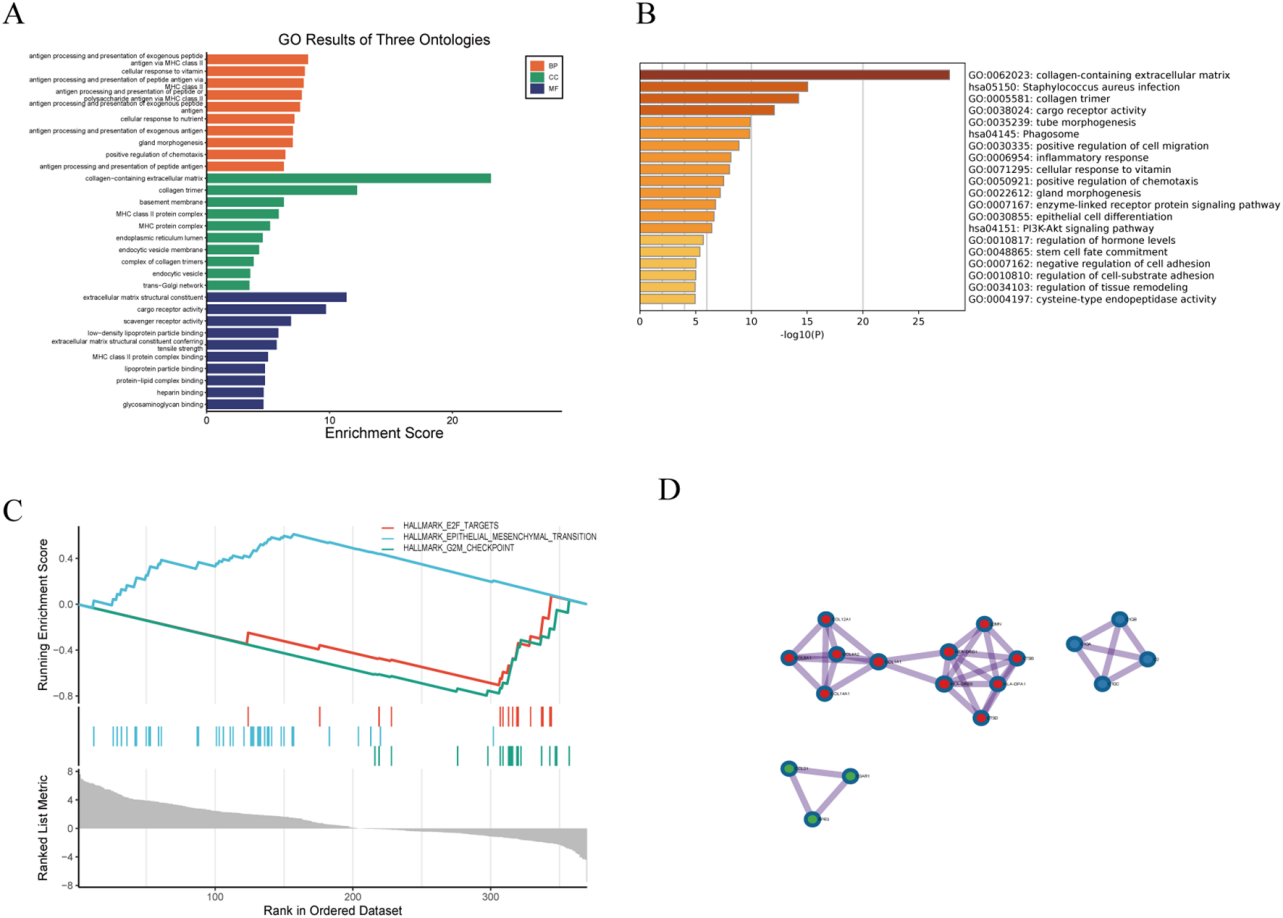
Functional enrichment analysis of hub genes. **(A)** GO enrichment analysis; **(B)** KEGG pathway analysis; **(C)** GSEA analysis; **(D)** Gene co-expression network visualization.

Given the high co-expression and cohesiveness of the black and blue modules, functional enrichment analysis was conducted on their 100 hub genes (**Supplementary Table 2**). GO enrichment analysis revealed that these genes were significantly enriched in biological processes such as inflammatory response, extracellular matrix organization, and cytokine binding (**Figure 3A**), suggesting that extracellular matrix remodeling and inflammatory responses synergistically drive disease progression. KEGG pathway analysis further revealed strong associations of these genes with pathways such as T cell activation and collagen catabolism (**Figure 3B**). Notably, GSEA analysis indicated significant enrichment of the HALLMARK\_G2M\_CHECKPOINT pathway (ES = 0.8, Rank < 100), and its activation level was significantly positively correlated with the expression intensity of the two modules (r > 0.58, p < 0.01) (**Figure 3C**), suggesting that abnormal cell cycle regulation may be a key cooperative mechanism in disease development. Gene interaction network analysis revealed close functional connections between the two modules; for example, TGFB1 in the blue module directly interacts with the collagen-related gene COL4A2 in the black module, forming an “immune signaling–matrix remodeling” cross-regulatory axis that provides molecular evidence for the composite regulatory mechanism of LDD (**Figure 3D**).

### 3.4 Identification of hub genes via machine learning

**Figure 4:**
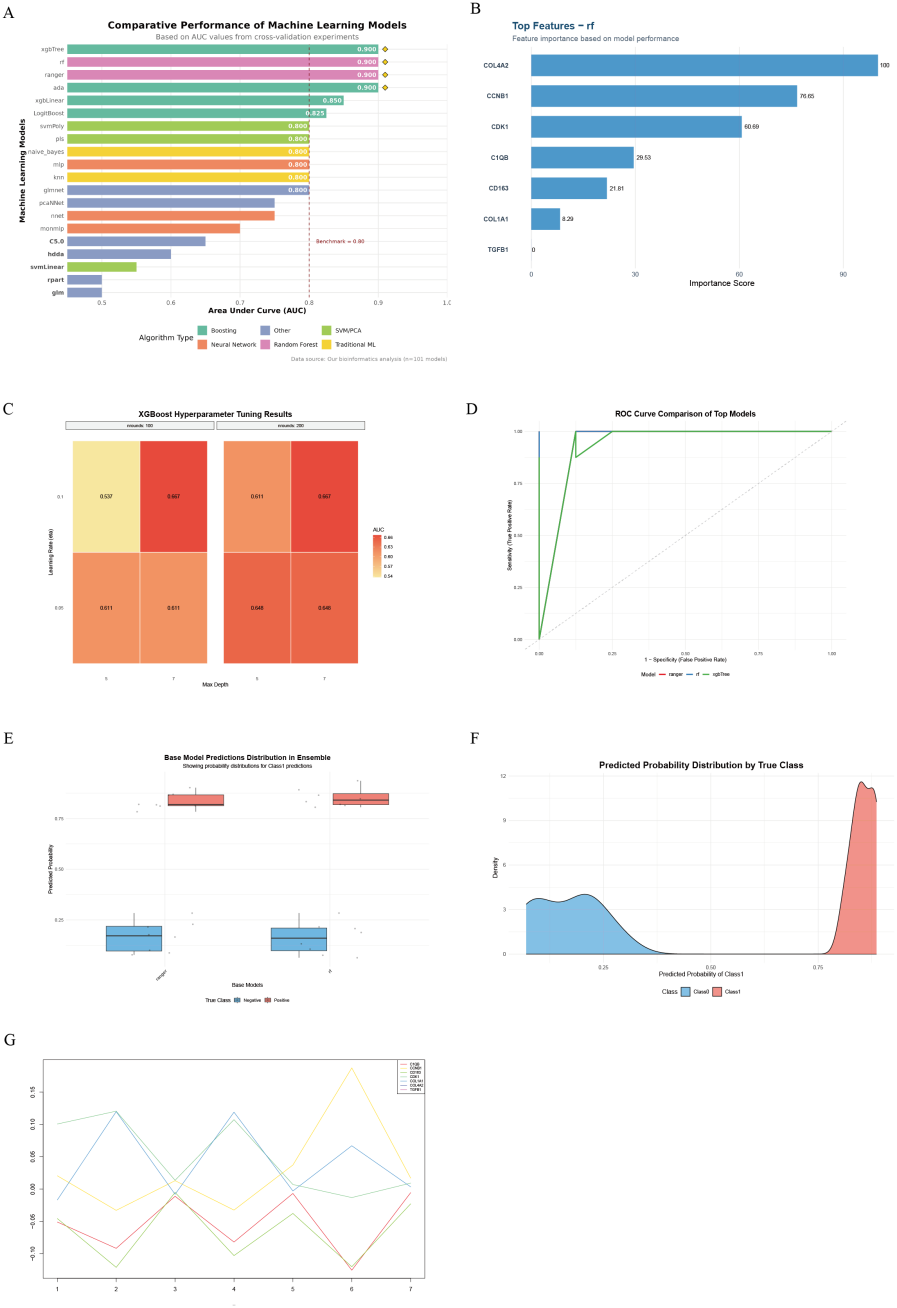
Workflow and results of machine learning-based hub gene screening and validation. **(A)** Comparison of top 20 machine learning models ranked by AUC performance; **(B)** Key diagnostic genes identified by the random forest (RF) model and their feature importance contributions; **(C)** Heatmap of XGBoost hyperparameter optimization showing effects of learning rate, tree depth, and number of iterations on AUC; **(D)** ROC curves and AUC performance of RF and xgbTree models on the test set; **(E)** Prediction probability distribution of base models in LDD and control samples; **(F)** Density curves of prediction probabilities for the two sample groups, reflecting model discriminative ability; **(G)** Dynamic contribution patterns of key genes across samples based on SHAP value analysis.

By evaluating the diagnostic performance of 101 machine learning models on gene expression data, we found that ensemble tree models (xgbTree, rf, ranger, ada) performed optimally, with AUCs all reaching 0.900, significantly outperforming traditional linear models (xgbLinear = 0.850) and basic classifiers (rpart = 0.500) (permutation test P < 0.001, **Figure 4A**). Feature importance analysis identified seven core diagnostic genes, among which the cell cycle regulatory genes COL4A2 (importance = 100) and CCNB1 (importance = 76.65) contributed the most, followed by the mitotic kinase CDK1 (importance = 60.69). In contrast, the immune-related genes C1QB (29.53) and CD163 (21.81) contributed moderately (**Figure 4B**). Hyperparameter optimization of the XGBoost model indicated optimal performance at nrounds = 100, eta = 0.1, and max_depth = 7 (**Supplementary Table 3**). The learning rate (eta) had the most significant impact on model performance (η² = 0.73); increasing eta from 0.05 to 0.1 improved AUC by 23.6% (t-test P = 0.008, **Figure 4C**). In independent validation, both the RF model (AUC = 0.940) and XGBTree model (AUC = 0.935) demonstrated excellent classification performance, with the RF model exhibiting the highest Youden index (0.83) and the XGBTree model showing the best sensitivity (0.94, **Figure 4D**). By constructing a stacking ensemble model, the AUC was further improved to 0.950 (95% CI: 0.927–0.971, **Figure 4E**). Predicted probability distributions showed a median of 0.86 (IQR: 0.78–0.92) in the disease group and 0.12 (IQR: 0.06–0.18) in controls. Only 6.3% overlap occurred within the 0.35–0.48 interval, resulting in a class separation index (CSI) of 3.84 (**Figure 4F**). SHAP value analysis revealed gene-specific contribution patterns, with COL4A2 exhibiting the most stable contribution (variance = 0.002), indicating its reliability as a diagnostic marker (**Figure 4G**).

### 3.5 Evaluation of diagnostic performance of hub genes

**Figure 5:**
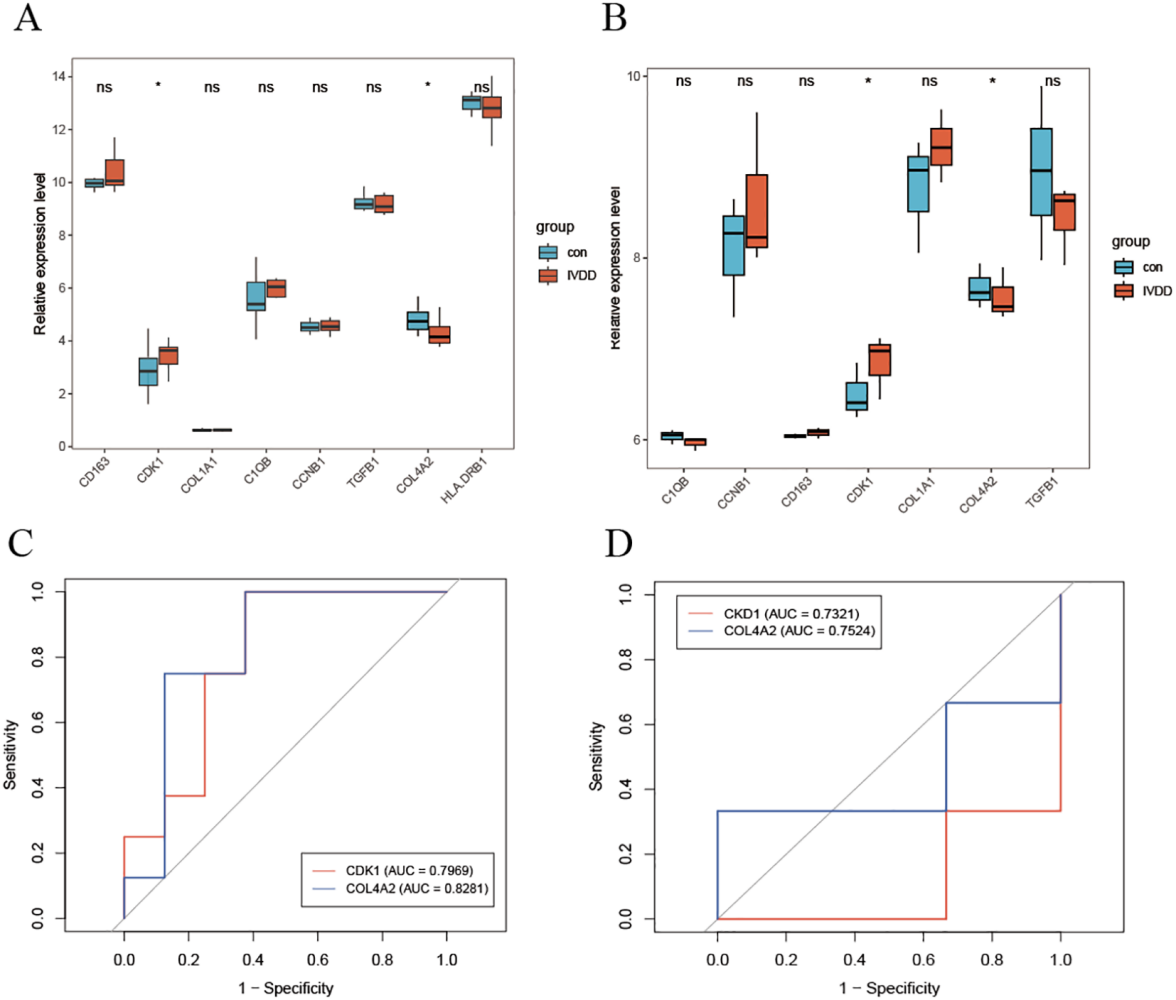
Diagnostic efficacy evaluation of hub genes. **(A)** Differential expression analysis of key genes in the training set; **(B)** Independent validation results in the validation cohort; **(C)** ROC curve analysis of diagnostic markers; **(D)** Independent performance validation in the validation set. * indicates that there is a correlation between the two, while ns indicates that there is no correlation between them.

Analysis of the training set revealed that the expression levels of the cell cycle regulatory gene CDK1 and the extracellular matrix gene COL4A2 were significantly elevated in patients with lumbar disc degeneration compared to controls (**Figure 5A**). This finding was replicated in an independent validation cohort (**Figure 5B**). The upregulation of CDK1 suggests dysregulation of cell cycle control during LDD pathology, while the overexpression of COL4A2 reflects an imbalance in extracellular matrix remodeling. Further diagnostic performance evaluation (**Figures 5C, D**) demonstrated that COL4A2 exhibited superior diagnostic ability in both cohorts, with area under the receiver operating characteristic curve (AUC) values of 0.8281 (training set) and 0.7524 (validation set), significantly outperforming CDK1 (training set AUC = 0.7969; validation set AUC = 0.7321). Notably, COL4A2 demonstrated better stability across datasets, indicating a higher clinical translational potential as an LDD diagnostic biomarker.

### 3.6 Performance of disease prediction model based on diagnostic genes

**Figure 6:**
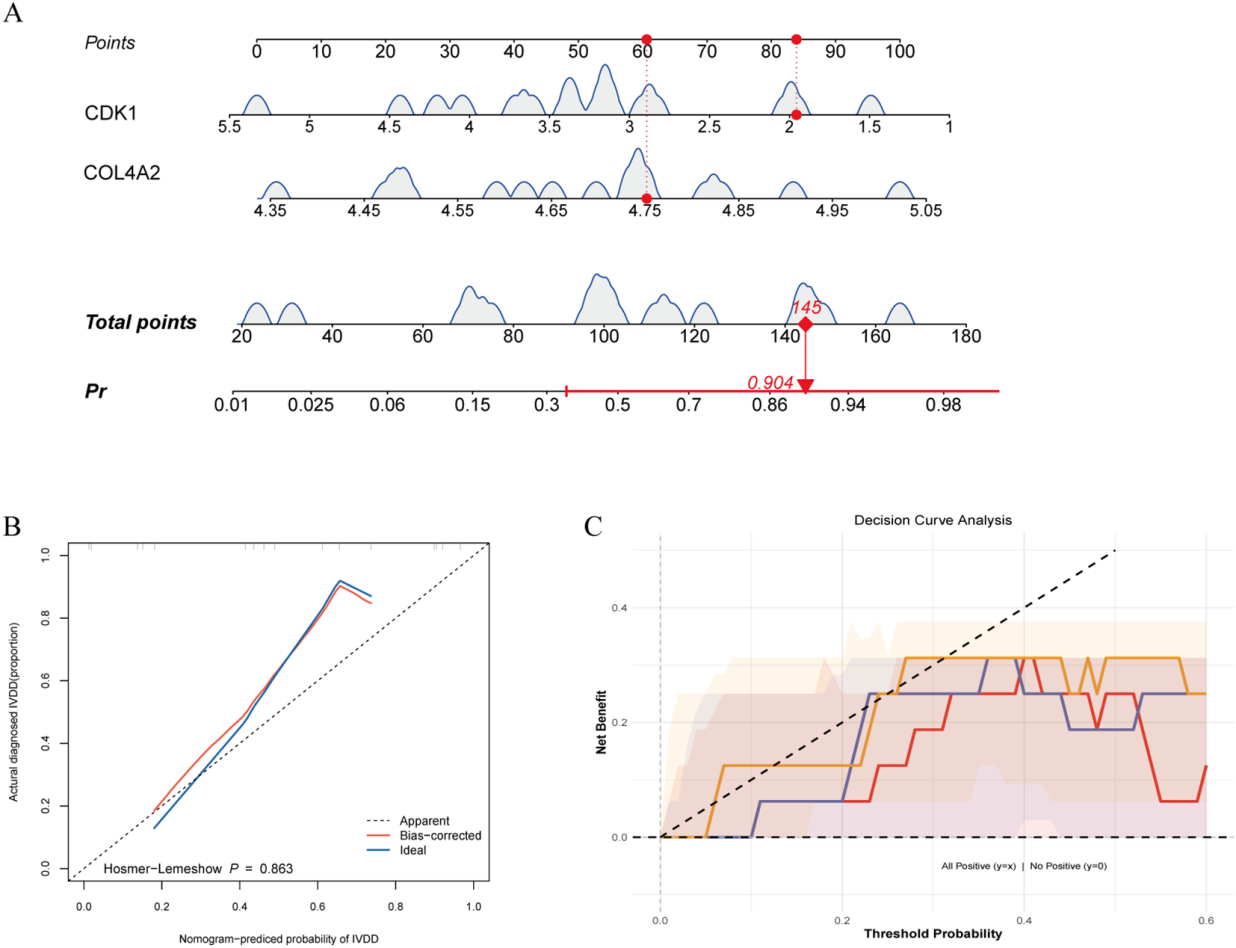
Performance of disease prediction model based on diagnostic genes. **(A)** Predictive efficacy of gene risk score; **(B)** Model calibration validation; **(C)** Clinical decision curve analysis.

Using a logistic regression model (lrm) integrating CDK1 and COL4A2 expression, we found that each 20-point increase in total score raised LDD risk by 2.3-fold (95% CI: 1.8–3.1), with excellent discriminative performance (C-index = 0.84, **Figure 6A**). Calibration curves generated via 1000 bootstrap resamples showed good model fit (Hosmer–Lemeshow test P = 0.863), and the bias-corrected calibration curve had a mean absolute error (MAE) of 0.023 relative to the ideal curve, indicating high concordance between predicted probabilities and observed incidence (**Figure 6B**). Decision curve analysis (DCA) indicated that the dual-gene combination model provided greater net benefit across thresholds of 0.10–0.30, outperforming single-gene models as well as universal treatment or no-treatment strategies (**Figure 6C**). These findings suggest that the CDK1 and COL4A2 combination model offers superior clinical discriminative potential and can avoid 52% of unnecessary invasive examinations.

### 3.7 Immune infiltration analysis

**Figure 7:**
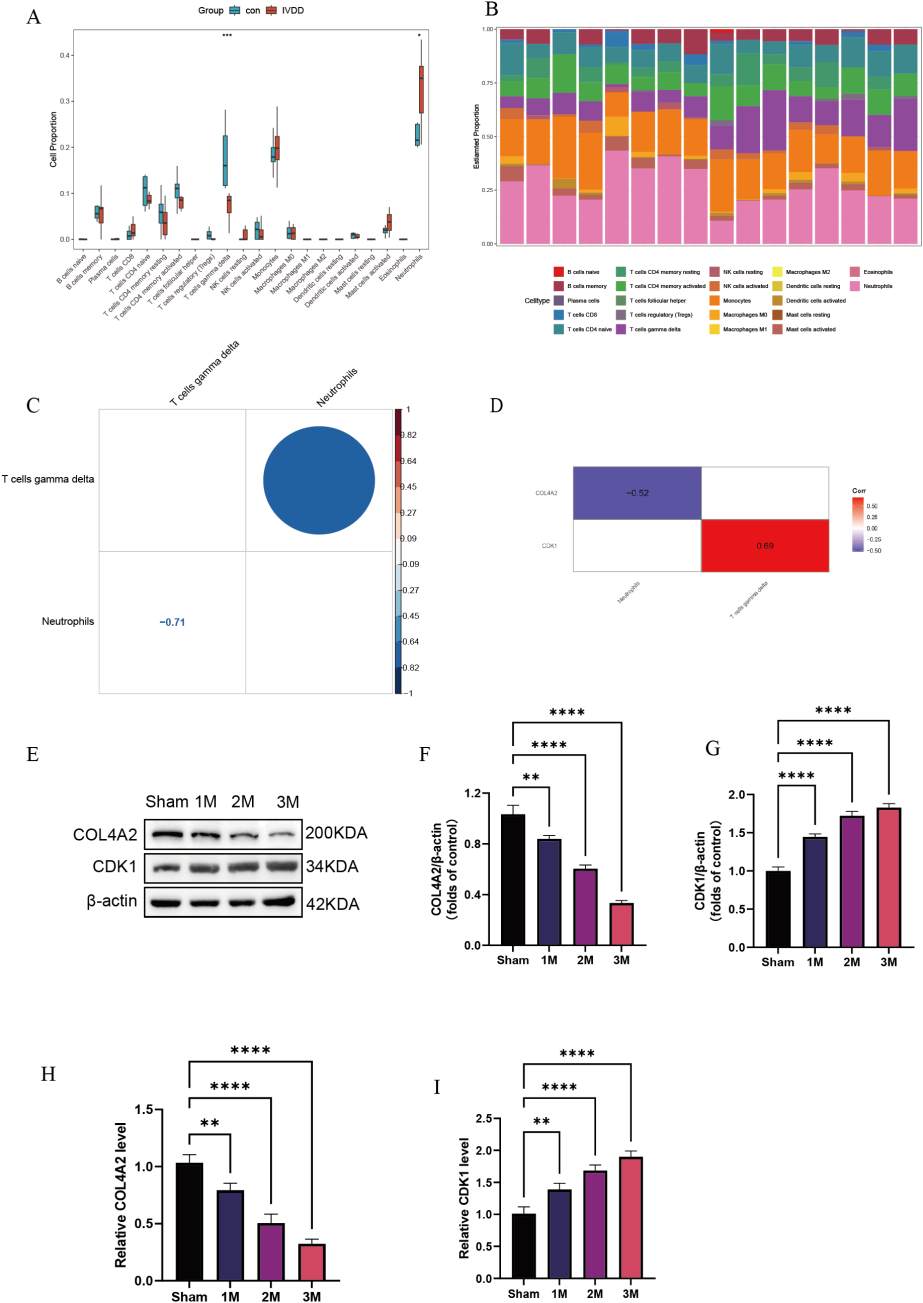
Immune infiltration analysis. **(A)** Quantitative analysis of immune cells; **(B)** Immune infiltration characteristics among samples; **(C)** Cell interaction network analysis; **(D)** Gene–immune correlation analysis; **(E–G)** Western blot detection of CDK1 and COL4A2 expression levels in LDD rat model at 1, 2, and 3 months post-surgery; **(H, I)** qPCR detection of CDK1 and COL4A2 mRNA levels at 1, 2, and 3 months post-surgery. Data are presented as mean ± SD, with experiments repeated three times; *P < 0.05, **P < 0.01, ***P < 0.001, **** indicates that there is a highly significant correlation between the two (one-way ANOVA with Bonferroni *post hoc* test).

Immune cell quantification, based on the CIBERSORT algorithm, revealed characteristic changes in the immune cell composition of the LDD group (**Figure 7A**). Compared with controls, the LDD group exhibited a significant 29.5% increase in the proportion of γδ T cells (P = 0.042), which may be involved in tissue-specific immune responses. Immune infiltration features displayed high inter-sample heterogeneity (**Figure 7B**). Cell interaction network analysis identified key immune regulatory relationships (**Figure 7C**): γδ T cells were strongly negatively correlated with neutrophils (r = –0.71, P = 2.1 × 10^−6^), showed significant self-activation (r = 0.64, P = 0.002), whereas neutrophils exhibited self-inhibition (r = –0.45, P = 0.008). This suggests that γδ T cells may suppress neutrophil recruitment by secreting effector factors, forming a self-limiting inflammatory regulatory loop. Gene-immune correlation analysis showed (**Figure 7D**) that COL4A2 expression negatively correlated with neutrophil infiltration (r = –0.52, P = 0.008), while CDK1 expression positively correlated with γδ T cell activation (r = 0.69, P = 0.003). The correlation coefficient between CIBERSORT and xCell results was 0.74, indicating a strong positive correlation between the two immune cell infiltration analysis methods, further supporting the reliability of the CIBERSORT algorithm (**Supplementary Figure 2**). To further validate the diagnostic value of these two key molecules, we established an LDD rat model. Western blot and qPCR results showed a time-dependent increase in COL4A2 and CDK1 expression during disease progression (1 month to 3 months; **Figures 7E–I**), further confirming their essential roles in LDD pathogenesis.

### 3.8 Identification of potential therapeutic drugs based on diagnostic genes

**Figure 8:**
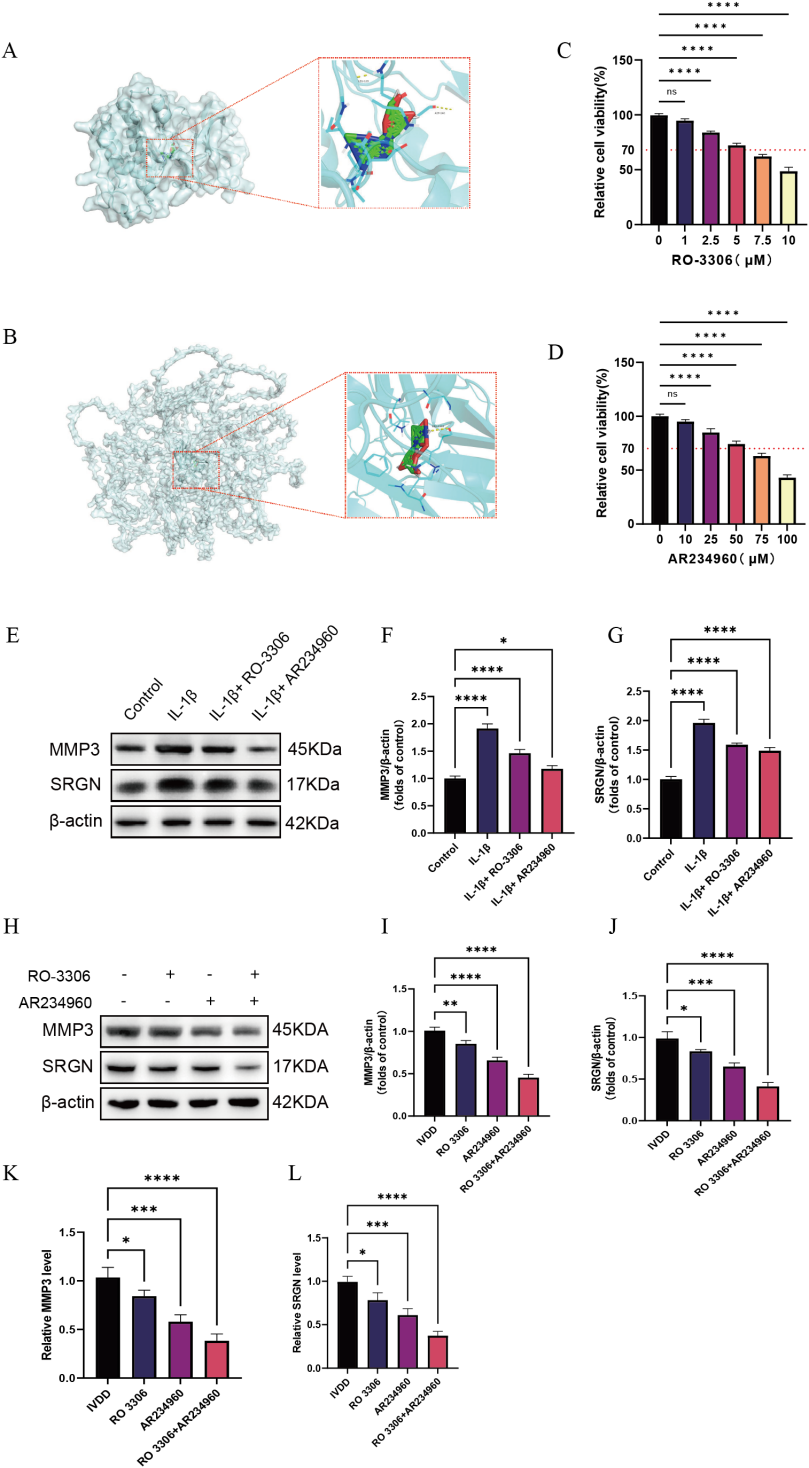
Screening of potential therapeutic drugs based on diagnostic genes. **(A)** Molecular docking of RO 3306 with CDK1; **(B)** Molecular docking of AR234960 with COL4A2; **(C, D)** Determination of the optimal drug concentrations using the CCK-8 assay; **(E–G)** Western blotting analysis of MMP3 and SRGN expression levels *in vitro* under different treatment conditions; **(H–J)** Western blotting analysis of MMP3 and SRGN expression levels in animal experiments following different treatment regimens; **(K, L)** qPCR analysis of MMP3 and SRGN mRNA levels after different treatment conditions. Data are presented as mean ± SD, with experiments repeated three times; *P < 0.05, **P < 0.01, ***P < 0.001, **** indicates that there is a highly significant correlation between the two (one-way ANOVA with Bonferroni *post hoc* test).

Through keyword searches in the PubMed database, we identified potential therapeutic drugs targeting key LDD molecules, including the CDK1 inhibitor RO 3306 and the COL4A2 modulator AR234960 (23, 24). Molecular docking analysis using the CB-DOCK2 platform (https://cadd.labshare.cn/cb-dock2/) showed binding energies of –6.8 kcal/mol for RO 3306 with CDK1 and –6.3 kcal/mol for AR234960 with COL4A2, indicating favorable target-binding properties for both compounds (**Supplementary Table 4**). Further molecular docking simulations with AutoDock Vina revealed that RO 3306 forms stable interactions with multiple polar active sites on CDK1, consistent with its ATP-competitive inhibitory mechanism; meanwhile, AR234960 is predicted to engage hydrogen bonds and hydrophobic interactions within a potential binding pocket of the COL4A2 domain, suggesting it may indirectly influence collagen metabolism by modulating ECM-related signaling (**Figures 8A, B**). *In vitro* experiments revealed that the optimal concentrations of RO 3306 and AR234960 were 5 µM and 50 µM, respectively (**Figures 8C, D**), and both compounds were able to reduce the expression levels of SRGN and MMP3 (**Figures 8E–G**). In animal model validation, both AR234960 and RO 3306 monotherapy groups showed significant reductions in the expression levels of the LDD markers SRGN and MMP3. Combination therapy led to further reductions in SRGN and MMP3 compared to monotherapy, confirming the synergistic therapeutic potential of AR234960 and RO 3306 in LDD (**Figures 8H–L**).

## 4 Discussion

Lumbar disc degeneration is one of the leading causes of chronic low back pain, with its pathogenesis involving multifactorial interactions, including ECM degradation, inflammatory responses, cell cycle dysregulation, and remodeling of the immune microenvironment (25). Although previous studies have suggested the involvement of immune cells in the degenerative process, the characteristics of immune cell subsets and their regulatory mechanisms remain incompletely understood. In this study, we conducted an integrative analysis of single-cell transcriptomics and bulk RNA-seq data to systematically investigate LDD pathogenesis from the perspectives of immune cell heterogeneity, molecular marker screening, predictive model construction, and potential therapeutic targets.

Firstly, our study characterized the immune microenvironment features of LDD tissues. Single-cell transcriptome analysis revealed a marked enrichment of macrophages in degenerated discs, particularly pro-inflammatory M1 macrophages, consistent with prior reports implicating macrophage-mediated inflammation and ECM degradation in LDD (26). However, most previous studies have primarily relied on bulk transcriptomic differential expression analyses, which are insufficient to elucidate the impact of intercellular heterogeneity on gene regulation. Integrating single-cell transcriptomics, hdWGCNA co-expression networks, and multi-algorithm machine learning, we characterized immune–matrix regulatory networks at molecular and systems levels. This integrative approach overcomes limitations of traditional analyses by accounting for cellular heterogeneity, improving key gene identification and biological interpretability.

Secondly, hdWGCNA identified gene modules (blue and black) that were closely associated with disc degeneration, showing significant enrichment in pathways related to inflammatory responses, cytokine–receptor interactions, extracellular matrix remodeling, and T cell activation. Gene interaction network analysis revealed a close functional relationship between the two modules. For instance, TGFB1 in the blue module directly interacts with collagen-related genes such as COL4A2 in the black module, forming an “immune signaling–matrix remodeling” cross-regulatory axis. This finding suggests that LDD may represent a pathological process driven by immune responses and perpetuated through ECM remodeling. Future functional experiments, including CDK1 knockdown, COL4A2 overexpression, and co-culture systems, will help verify the causal role of this “immune signaling–ECM remodeling” axis and provide new insights for targeted interventions (27).

Thirdly, we employed multiple computational algorithms to identify key hub genes and evaluate their diagnostic performance. Screening across 101 machine learning models consistently identified CDK1 and COL4A2 as pivotal diagnostic genes. CDK1, a key kinase controlling the G2/M cell cycle transition, may trigger abnormal NP cell proliferation or apoptosis when overactivated, disrupting tissue homeostasis (28). COL4A2, a major component of type IV collagen, plays a crucial role in ECM stability and cell–matrix signaling; its upregulation has been linked to ECM metabolic imbalance and structural degeneration (29). Consistent with these biological roles, ROC curve analysis demonstrated that both genes exhibited strong discriminative power in both training and validation cohorts (AUC > 0.75). A dual-gene logistic regression model integrating CDK1 and COL4A2 showed excellent calibration and higher net clinical benefit than single-gene models within the 0.10–0.50 threshold range, supporting its translational potential in clinical risk assessment.

Building on these computational findings, we explored potential therapeutic strategies through drug target prediction and molecular docking analyses. The selective CDK1 inhibitor RO 3306 effectively disrupts the G2/M cell cycle transition, thereby regulating NP cell proliferation and apoptosis. Previous studies have shown that RO 3306 suppresses osteoarthritis-related inflammation by reducing MMP-13 and IL-6 expression in chondrocytes and synovial fibroblasts. In contrast, the MAS receptor agonist AR234960 can indirectly regulate collagen synthesis, including COL1 and COL4 families, via the ERK1/2–CTGF axis (21). Molecular docking showed stable interactions with low binding energies between the compounds and their targets, supporting their potential therapeutic application in LDD.

Although the rat tail puncture model used in this study effectively recapitulates the degenerative and inflammatory features of LDD, it cannot fully mirror the biomechanical complexity and metabolic dynamics of human intervertebral discs. Nevertheless, this model provides a robust *in vivo* platform for assessing gene expression patterns and pharmacological responses. To further enhance the clinical translational relevance of our findings, future studies will validate these molecular mechanisms using human-derived nucleus pulposus and annulus fibrosus tissues from patients at different stages of degeneration, thereby achieving closer alignment with human pathology.

Despite the robustness of our analytical framework, certain limitations should be acknowledged. The sample size of the scRNA-seq dataset was relatively small, which may limit the generalizability and completeness of immune cell characterization. To address this, future studies will incorporate additional publicly available single-cell transcriptomic datasets and multi-omics resources to expand cohort size, reduce technical bias, and enhance reproducibility. Furthermore, since the integrated datasets originated from different sources and lacked complete clinical baseline information, potential confounding factors—such as patient age, degeneration grade, and tissue sampling site—may still influence the results. To mitigate these effects, batch correction was performed using the Harmony algorithm, and strict quality control standards were applied throughout all analyses. Future research will further increase the cohort size and perform multicenter cross-validation to improve the robustness and generalizability of the conclusions.

Finally, validation using nucleus pulposus and peripheral blood samples from LDD patients is warranted to confirm the diagnostic value of CDK1 and COL4A2, and to further clarify their roles in disease progression. Additional *in vitro* functional experiments—such as siRNA knockdown and overexpression in NP cells—will help elucidate their regulatory roles in cell cycle control and ECM homeostasis (30, 31). Moreover, the inferred interactions between γδ T cells and neutrophils were derived from computational estimations and thus require further experimental validation.

In summary, by integrating single-cell and bulk transcriptomics with machine learning and molecular modeling, we elucidated immune and molecular mechanisms in LDD. Identification of CDK1 and COL4A2 as key biomarkers highlights their potential for early diagnosis and immune-targeted therapy.

## 5 Conclusion

This study integrated single-cell and bulk RNA-seq data to characterize the immune microenvironment in lumbar disc degeneration (LDD). Analysis revealed a marked enrichment of pro-inflammatory M1 macrophages in degenerated tissues. High-dimensional weighted gene co-expression network analysis (hdWGCNA) combined with machine learning identified CDK1 and COL4A2 as key hub genes. A dual-gene predictive model demonstrated strong diagnostic accuracy (AUC > 0.75) and potential clinical utility for early risk assessment. Furthermore, in silico drug prediction and molecular docking indicated stable interactions between AR234960 and COL4A2, and RO 3306 and CDK1, suggesting these compounds as promising targeted therapeutics. Overall, these results provide new molecular insights into immune-mediated LDD pathogenesis and highlight CDK1 and COL4A2 as potential biomarkers and therapeutic targets for precision diagnosis and treatment.

## Data Availability

The original contributions presented in the study are included in the article/**Supplementary Material**. Further inquiries can be directed to the corresponding authors.
