# Supplementary figures and images for: Integration of single-cell sequencing and machine learning identifies key macrophage-associated genetic signatures in lumbar disc degeneration
Source: Front Immunol. 2025 Dec 2;16:1671961. doi: 10.3389/fimmu.2025.1671961 (PMC12705385; doi:10.3389/fimmu.2025.1671961)

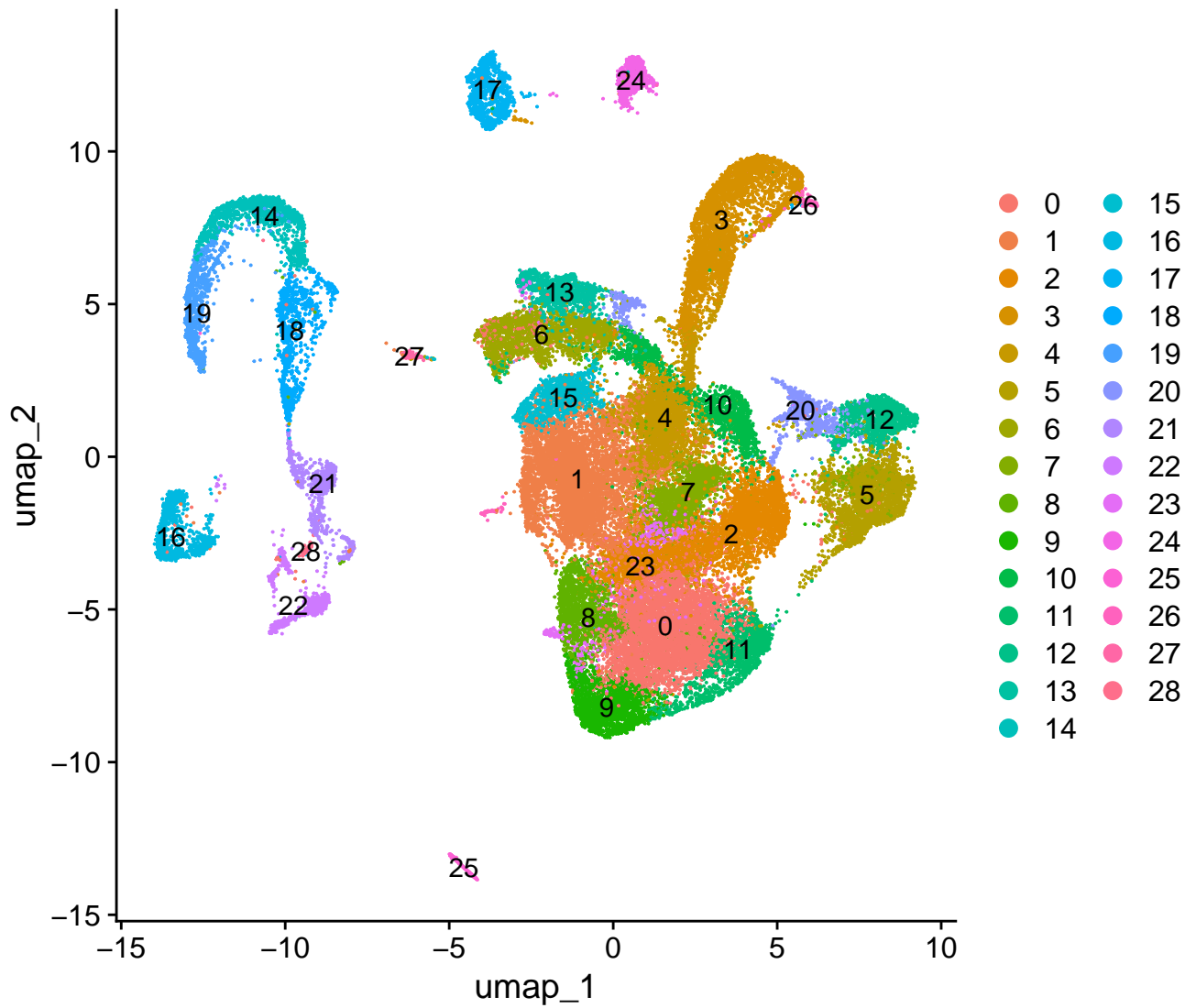

Supplement: Supplementary file 1 [file DataSheet1.pdf]

# Correlation between CIBERSORT and xCell results

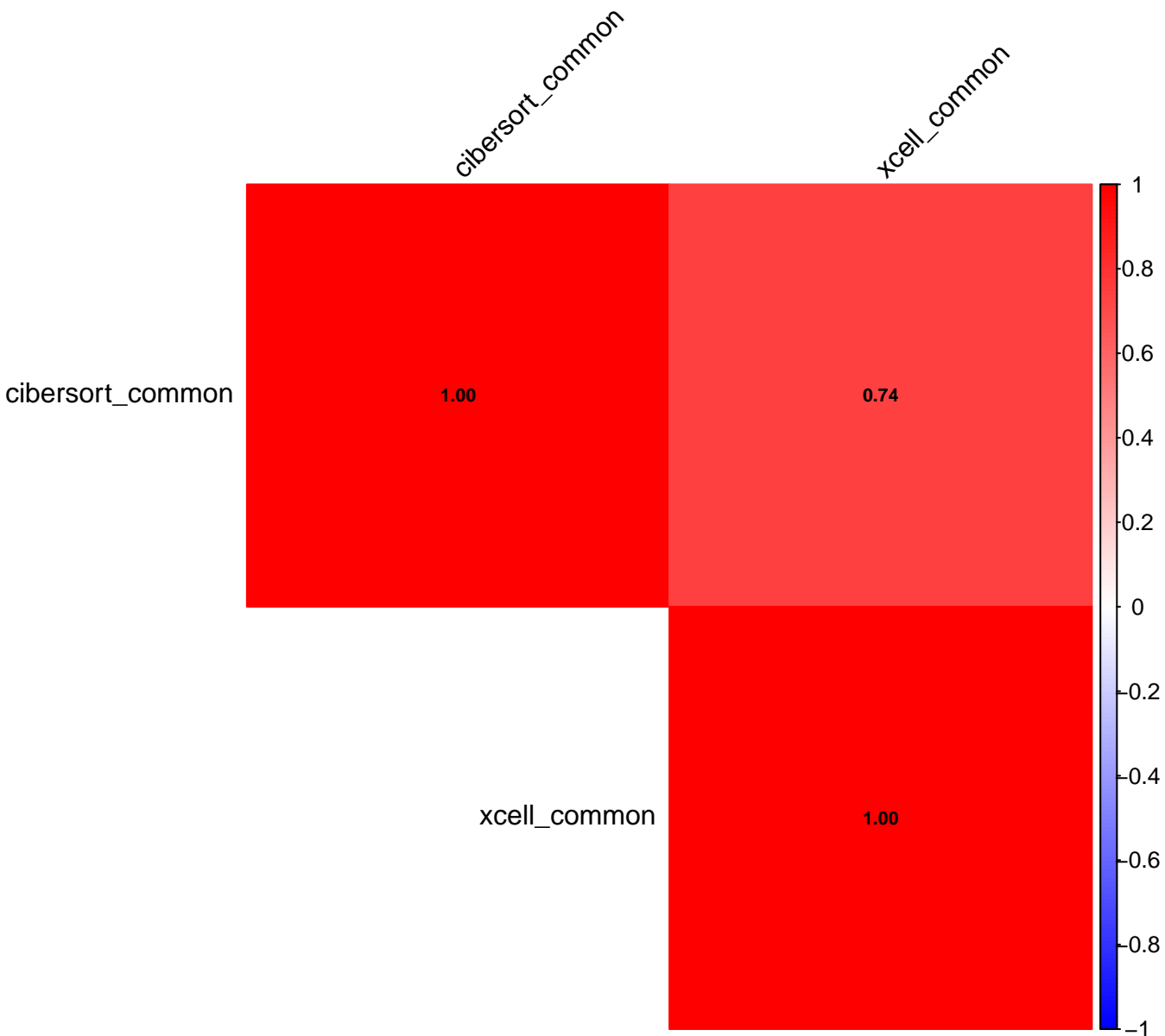

Supplement: Supplementary file 2 [file DataSheet2.pdf]
